# Development of an Amplicon Nanopore Sequencing Strategy for Detection of Mutations Conferring Intermediate Resistance to Vancomycin in Staphylococcus aureus Strains

**DOI:** 10.1128/spectrum.02728-22

**Published:** 2023-01-23

**Authors:** Abraham G. Moller, Robert A. Petit, Michelle H. Davis, Timothy D. Read

**Affiliations:** a Division of Infectious Diseases, Department of Medicine, Emory University, Atlanta, Georgia, USA; b Microbiology and Molecular Genetics (MMG), Graduate Division of Biological and Biomedical Sciences (GDBBS), Emory University, Atlanta, Georgia, USA; c Theiagen Genomics, Highlands Ranch, Colorado, USA; University at Albany, State University of New York

**Keywords:** *Staphylococcus aureus*, amplicon sequencing, antibiotic resistance, clinical microbiology, diagnostics, genomics, nanopore sequencing, vancomycin resistance, vancomycin-intermediate *Staphylococcus aureus*

## Abstract

Staphylococcus aureus is a major cause of bacteremia and other hospital-acquired infections. The cell-wall active antibiotic vancomycin is commonly used to treat both methicillin-resistant (MRSA) and sensitive (MSSA) infections. Vancomycin intermediate S. aureus (VISA) variants can arise through *de novo* mutations. Here, we performed pilot experiments to develop a combined PCR/long-read sequencing-based method for detection of previously known VISA-causing mutations. Primers were designed to generate 10 amplicons covering 16 genes associated with the VISA phenotype. We sequenced amplicon pools as long reads with Oxford Nanopore adapter ligation on Flongle flow cells. We then detected mutations by mapping reads against a parental consensus or known reference sequence and comparing called variants against a database of known VISA mutations from laboratory selection. Each amplicon in the pool was sequenced to high (>1,000×) coverage, and no relationship was found between amplicon length and coverage. We also were able to detect the causative mutation (*walK* 646C>G) in a VISA mutant derived from the USA300 strain (N384-3 from parental strain N384). Mixing mutant (N384-3) and parental (N384) DNA at various ratios from 0 to 1 mutant suggested a mutation detection threshold of the average minor allele frequency (6.5%) at 95% confidence (two standard errors above mean mutation frequency). The study lays the groundwork for direct S. aureus antibiotic resistance genotype inference using rapid nanopore sequencing from clinical samples.

**IMPORTANCE** Bacteremia mortality is known to increase rapidly with time after infection, making rapid diagnostics and treatment necessary. Successful treatment depends on correct administration of antibiotics based on knowledge of strain antibiotic susceptibility. Staphylococcus aureus is a major causative agent of bacteremia that is also commonly antibiotic resistant. In this work, we develop a method to accelerate detection of a complex, polygenic antibiotic resistance phenotype in S. aureus, vancomycin-intermediate resistance (VISA), through long-read genomic sequencing of amplicons representing genes most commonly mutated in VISA selection. This method both rapidly identifies VISA genotypes and incorporates the most comprehensive database of VISA genetic determinants known to date.

## INTRODUCTION

Staphylococcus aureus is a commensal carried by 30% of humans (1) and a nosocomial pathogen that causes diverse pathologies. Many clinical strains are antibiotic resistant, with methicillin resistance (MRSA) first reported in the 1960s (2). The glycopeptide vancomycin, first released in 1958, has long been used for the treatment of severe MRSA infections (3). However, resistance to vancomycin has emerged in S. aureus through two distinct genetic processes. High-level vancomycin resistance (VRSA), acquired through horizontal acquisition of the *van*A gene, though detected in S. aureus in 2002 (4, 5), fortunately remains rare. Alternatively, vancomycin-intermediate resistance (VISA), the focus of this study, arises through *de novo* chromosomal mutations under antibiotic selection that are more often encountered in the clinic than VRSA (3).

VISA is defined by a vancomycin MIC between 4 and 8 μg/mL, while heterogeneous VISA (hVISA) strains have a vancomycin MIC between 2 and 4 μg/mL (the VRSA MIC is >16 μg/mL). Mutations associated with VISA are found in multiple sites in several key genes (6). The VISA phenotype includes a thickened cell wall, reduced autolysis, increased capsule, and increased d-alanylation of teichoic acids (3). The thickened VISA cell wall both reduces vancomycin diffusion and contains more free d-alanyl-d-alanine that can bind vancomycin, leading to reduction of vancomycin reaching the site of cell wall synthesis (the septum) and, thus, reduced sensitivity to the antibiotic (3). VISA strains furthermore have been shown to have reduced vancomycin susceptibility *in vivo* (3, 7) and to be associated with persistent bacteremia in clinical studies (3, 8–10). Despite its prevalence and significance, current VISA detection methods remain laborious and time-consuming (3, 11–13). VISA detection typically requires broth microdilution determination of MIC, while hVISA detection requires the more complex population analysis profile-area under the curve (PAP-AUC) assay (3). MIC and Etest assays require at least 24 h, and the PAP-AUC assay requires 48 h of incubation before resistance determination (3). Improving VISA treatment will thus require more rapid detection methods reducing or eliminating the time taken culturing.

Nanopore-based sequencing technologies offer a quicker alternative to culture-based tests for antibiotic resistance. DNA can be sequenced by pulling it through a protein nanopore, measuring the corresponding changes in current, and converting this current trace into a DNA sequence (14). While it has a higher per-base error rate (~10%) than short-read sequencing (e.g., Illumina), nanopore sequencing not only results in longer read length relative to other sequencing technologies (over 1 kb per read) but is also faster; sequencing data can be collected in real time, as early as the beginning of the sequencing run. Improvements in basecalling as well as pore chemistry itself have also led to reduced error rates, addressing its major weakness (15).

Recent studies have demonstrated that nanopore sequencing can detect antibiotic resistance genes in metagenomic samples (16–19) and distinguish antibiotic-sensitive from resistant strains potentially faster than culture-based methods, which typically require overnight growth (20, 21). A method for distinguishing carbapenem-resistant from -sensitive Klebsiella pneumoniae through 16S rRNA present in culture under imipenem treatment requires only 4 h of culturing (20). Another method that extrapolates antibiotic resistance from pneumococcal sequence type takes only 5 min (21). One nanopore sequencing method has been developed for direct detection of resistance-causing mutations in sexually transmitted infections from PCR amplicons (22), while other amplicon-targeted nanopore sequencing methods have focused on Mycobacterium tuberculosis drug resistance (23–26). Resistance mutations have also been detected in metagenomic nanopore sequence data collected from urine containing Neisseria gonorrhoeae (27). Additionally, genomic prediction of antibiotic resistance must still be calibrated thoroughly against culture-based methods (28).

Here, we present a pilot version of an approach to rapidly determine likely vancomycin-intermediate Staphylococcus aureus (VISA)-conferring mutations in S. aureus strains or clinical samples by coupling PCR and nanopore sequencing. This strategy offers a potential improvement on the current state of the art (e.g., quantitative PCR [qPCR] for resistance genes or mutations, culture-based testing) because there are many possible mutations in many genes that may cause VISA, including mutations we have never seen before, which makes sequencing followed by bioinformatic mutation detection and interpretation necessary. To our knowledge, this is the first study aimed at detecting mutations (single-nucleotide polymorphisms [SNPs] and small indels) linked to a polygenic antibiotic resistance phenotype through nanopore sequencing. We developed a PCR scheme to amplify 10 regions containing the 16 genes (*walR*, *walK*, *rpoB*, *graR*, *graS*, *vraF*, *vraG*, *stpI*, *vraR*, *vraS*, *agrA*, *sarA*, *clpP*, *ccpA*, *prsA*, and *yvqF*) most likely to contain VISA-conferring mutations based on previous work (6). We then developed a culture-free method to detect VISA strains using sequencing, reference-based alignment, and comparison to a database of known VISA-conferring mutations, which can be performed in less time than the 24+ hours necessary for culture-based detection. As a proof of principle, we have sequenced VISA amplicons from a parent strain (N384) and one mutant (N384-3) to detect VISA-conferring mutations. We also detected the mutation when its abundance was as low as 10% relative to the parent and detected VISA mutations directly from clinical VISA strains.

## RESULTS

### Sample isolation and PCR.

We amplified VISA regions of interest from DNA extracted from isolated bacterial cultures (see Fig. S1 in the supplemental material). We decided to forego development of blood-based extraction at this point to concentrate on steps 2 to 4 as outlined in Materials and Methods. We used DNA extracted from bacterial cultures with the modified Qiagen DNeasy blood and tissue minikit protocol to serve as a PCR template in subsequent experiments (extracting DNA from mixed N384 and N384-3 cultures). From these templates (diluted to 0.2 ng/μL), we amplified all 10 PCR amplicons (lengths in Table S1) to similar relative concentrations (70 ng/μL).

### Sequencing and mutation detection: limit of detection approaches average minor allele frequency.

**FIG 1:**
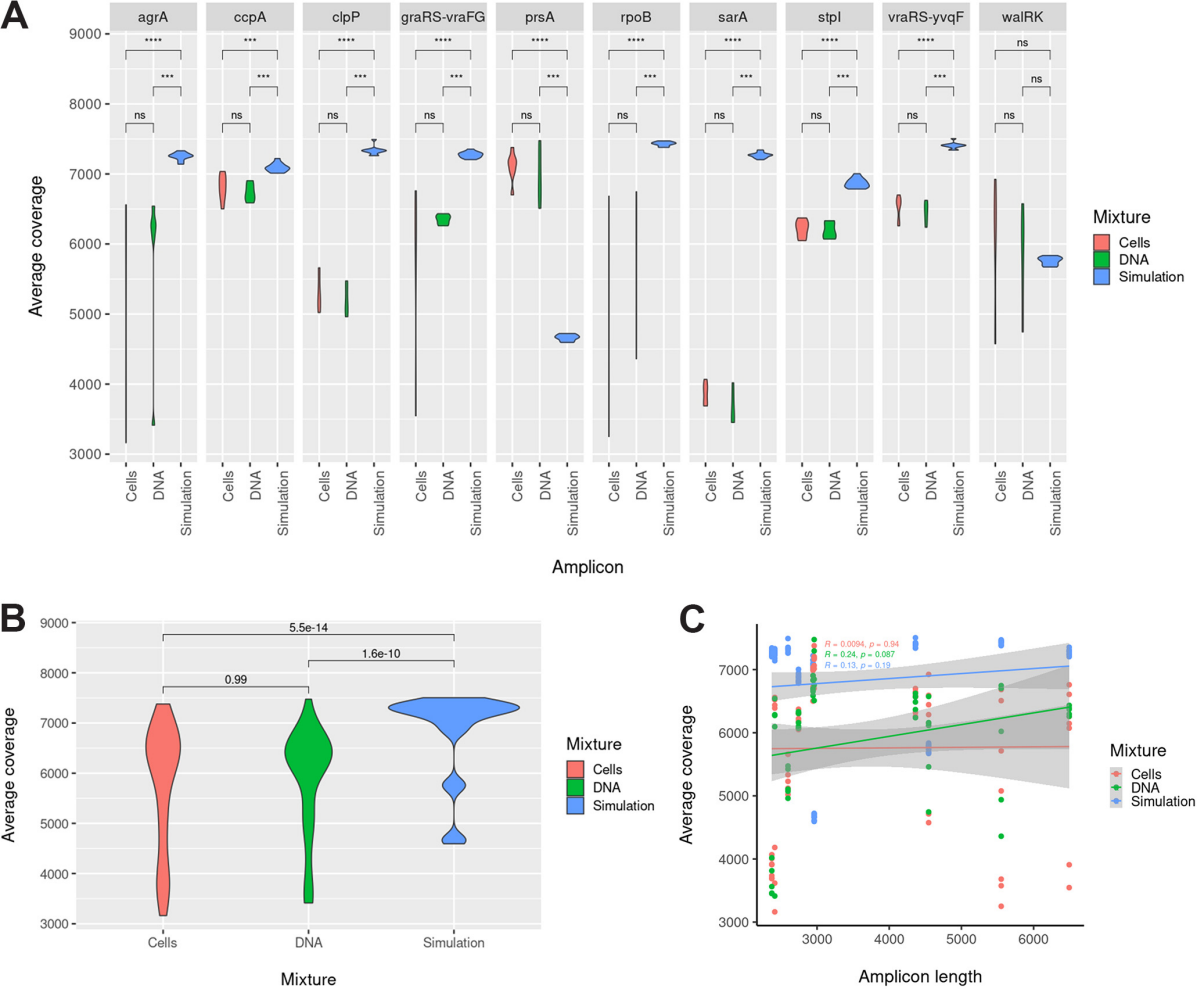
Gene coverage analyses for simulation (blue), DNA (amplicon; green), and cell (culture; red) parent/mutant mixtures. Average coverage was calculated for each gene and condition based on SAMtools mpileup counts of aligned bases in each gene. (A to C) Coverage distributions represent average coverages for all samples separated by mixture type (simulation, DNA, or cells) and amplicon (A) or just mixture type (B and C). (A) Coverage distribution for each of the 10 amplicons visualized as a violin plot; coverage for each amplicon is compared among mixture sets with a nonparametric Wilcoxon test. (B) Coverage distribution for each mixture set (simulation, DNA, or cell) visualized as a violin plot; coverage is compared between mixture sets with a nonparametric Wilcoxon test. (C) Coverage distribution against amplicon length for each mixture set presented with correlation and *P* value.

To evaluate the limit of detection of our assay, we prepared three different mixes of parental VSSA (N384) and VISA (N384-3) mutant DNA: (i) culture mixtures, (ii) DNA amplicon mixtures, and (iii) simulated amplicon sequencing read mixes. Culture (cell) and DNA amplicon mixtures had similar coverages to each other but not to simulated DNA read mixtures based on nonparametric Wilcoxon tests (Fig. 1A and 1B; *P* = 5.5e-14 and 1.6e-10, relative to culture and DNA mixtures, respectively, in Fig. 1B). The same pattern was present at a per-amplicon level (Fig. 1A), except for *walRK*, where all three sets overlapped, and *prsA*, where the simulated coverage was significantly lower than that of either (*P* = 6.3e-05 and 4.6e-4, relative to culture and DNA mixtures, respectively). In all cases (cell, DNA, and simulated mixtures), coverage was in excess of 3,000-fold, which is well above the inverse of the error rate (~1/0.05 or 20-fold), suggesting coverage is high enough to compensate for at least random errors. Additionally, in all cases, there was no significant relationship between amplicon length and amplicon coverage (Fig. 1C; *P* = 0.94 for cell, *P* = 0.087 for DNA, and *P* = 0.19 for simulated mixtures).

**FIG 2:**
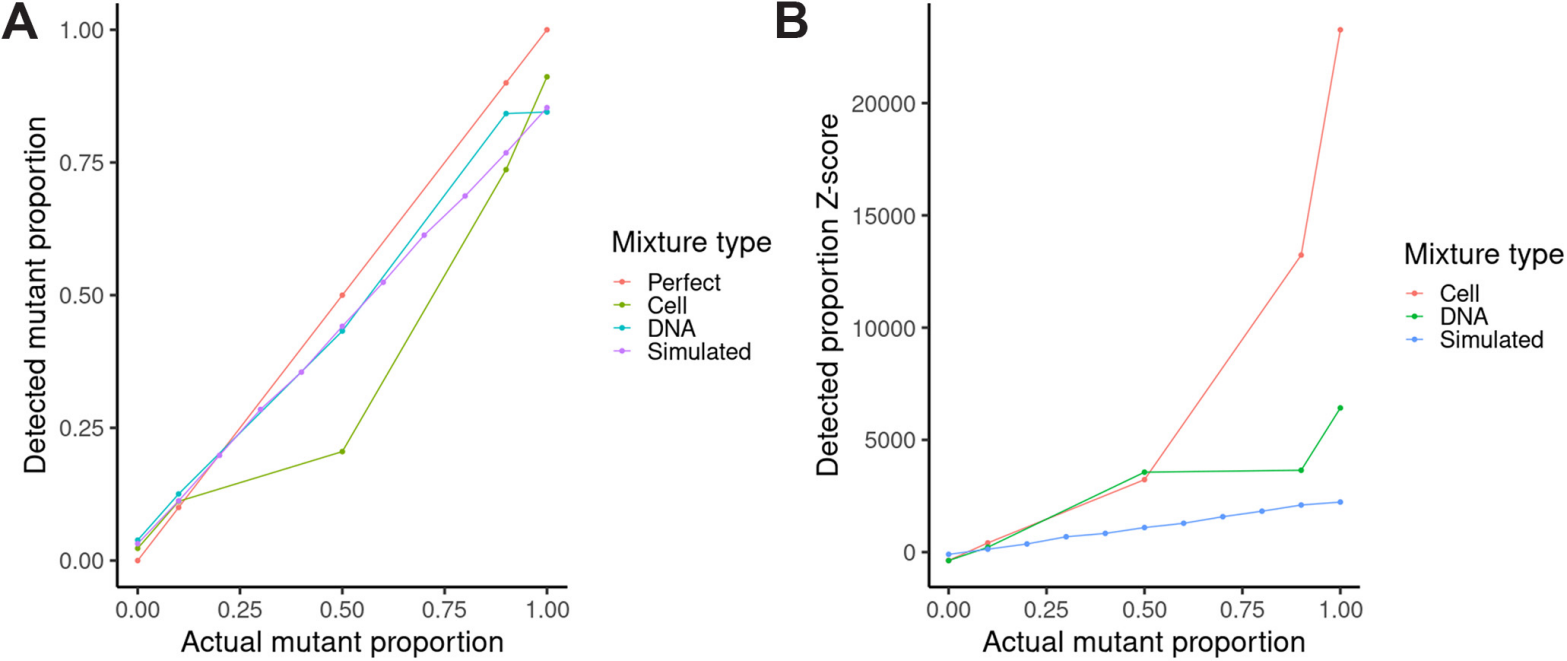
Evaluating the limit of detection over a range of mutant/parent mixes (cultures, amplicon DNA, and simulated sequence reads). (A) Introduced mutant proportion (*x* axis) versus detected mutation proportion (*y* axis) for culture (olive-green), amplicon DNA (blue-green), or simulated (purple) sequence read mixtures along with the diagonal expected if the introduced mutant proportion matched the detected mutation proportion (defined here as a “perfect” correlation; red). (B) Introduced mutant proportion (*x* axis) versus detected mutation Z-score (*y* axis) for cell (red), amplicon DNA (green), or simulated (blue) sequence read mixtures.

Given that we achieved high coverage for all amplicons in all cases, we proceeded to call mutations in each mixture (Table 1) and analyze the detected mutation frequency relation with mutant proportions in the mixture (Fig. 2). Among the simulated mixtures, Medaka detected only the N384-3 *walK* 646C>G mutation at a 60% mutant proportion in the mutant/parent mixture or higher, while the Burrows-Wheeler Aligner (BWA)/BCFtools detected the mutation at a proportion of 50% mutant or higher (Table 1). For the cell and DNA mixtures, on the other hand, Medaka detected this mutation at a 50% mutant proportion or higher, while BWA/BCFtools detected it at 10% mutant or higher (Table 1). This suggests the standard variant calling pipeline (BWA/BCFtools) was more sensitive with respect to mutant abundance than the faster Medaka. In addition, BCFtools had consistently lower coverage thresholds for detecting the *walK* mutation than Medaka when subsampling read data sets (Table 2). We also found that over 300-fold coverage was achieved within 10 min of sequencing in all mixtures (Table S2). Regarding the correlation between introduced and detected mutant proportions, we found the greatest deviation from a perfect correlation with the culture mixtures (Fig. 2A). At 50% mutant, the detected mutant proportion was below that detected from all other mixtures at the same introduced mutant proportion. We hypothesize that differences in DNA extraction efficiency between the VISA mutant and VSSA strain explain this deviation, as the thicker VISA cell walls would make DNA harder to extract, leading to a lower detected mutation proportion than expected based on how the cultures were mixed.

**TABLE 1:** Mutation calls (whether *walK* 646C>G was called) by BWA/BCFtools and Medaka for simulated, amplicon DNA, and culture mixtures

Proportion N384/N384-3	Simulated (NanoSim)		Amplicon DNA		Culture	
	BWA/BCFtools	Medaka	BWA/BCFtools	Medaka	BWA/BCFtools	Medaka
100% N384	No	No	No	No	No	No
90% N384	No	No	Yes	No	Yes	No
10% N384-3						
80% N384	No	No				
20% N384-3						
70% N384	No	No				
30% N384-3						
60% N384	No	No				
40% N384-3						
50% N384	Yes	No	Yes	Yes	Yes	No
50% N384-3						
40% N384	Yes	Yes				
60% N384-3						
30% N384	Yes	Yes				
70% N384-3						
20% N384	Yes	Yes				
80% N384-3						
10% N384	Yes	Yes	Yes	Yes	Yes	Yes
90% N384-3						
100% N384-3	Yes	Yes	Yes	Yes	Yes	Yes

**TABLE 2:** Minimum coverage required to call *walK* 646C>G mutation with BWA/BCFtools and Medaka in mutants or mutant mixtures or minimum coverage required to exclude mutation from wild-type samples for simulated, amplicon DNA, and culture mixtures^*a*^

Proportion N384/N384-3	Simulated (NanoSim) (×)		Amplicon DNA (×)		Culture (×)	
	BWA/BCFtools	Medaka	BWA/BCFtools	Medaka	BWA/BCFtools	Medaka
100% N384	0	0	200 (exclude C > T)	NA	0 (C > T at 10)	NA
90% N384	5	5	10	NA	150	NA
10% N384-3						
80% N384	5	NA				
20% N384-3						
70% N384	10	NA				
30% N384-3						
60% N384	5	5				
40% N384-3						
50% N384	2	5	20	80	5	20
50% N384-3						
40% N384	2	2				
60% N384-3						
30% N384	2	2				
70% N384-3						
20% N384	3	20				
80% N384-3						
10% N384	2	2	2	2	3	3
90% N384-3						
100% N384-3	10	10	2	2	2	2

a Cases in which the mutation was never detected are listed as NA.

We also examined the Z-score for each mixture and each set to determine the limit of detection based on the average and standard error of inherent sequencing variation found in the alignments (Fig. 2B). In all cases, average minor (nonconsensus) allele frequency was roughly 6.5%, and tested mutant proportions above this threshold were more than two standard errors (in fact, at least 100 standard errors) above the mean. Z-scores were highest for cell mixtures among the sets of mixtures evaluated (DNA and simulated reads). This suggests that the limit of detection based on our method is close (standard errors of minor allele frequency were roughly 1e-4) to the average minor allele frequency.

### Successful identification of VISA-associated mutations in a clinical VISA strain.

To show our assay could identify VISA mutations in clinical strains, we sequenced two known clinical strains—one VSSA (EUH15) and one VISA (107)—characterized in a previous study. Strain 107 is known to contain four missense mutations in VISA-associated genes (WalK A243T at 26374, GraR E15K at 708287, VraG T217I at 711484, VraS A314V at 1947464) relative to the N315 reference, while strain EUH15 contains none of these four. Mutation calling results from two methods (BWA/BCFtools and Medaka) are shown in Table 3. The first method (BWA/BCFtools) identified all four mutations in strain 107 but not strain EUH15, but the second method (Medaka) identified only three (in *walK*, *graR*, and *vraS*). This indicates that we can distinguish VISA from VSSA in at least two unknown clinical strains with our assay and bioinformatic pipeline. Standard variant calling (BWA/BCFtools) outperformed rapid variant calling (Medaka) in coverage sensitivity as we observed in our limit of detection study. As noted for the N384 parent/mutant mixtures, both Medaka and BCFtools detected VISA mutations of interest at 5× coverage or less (Table 4), except for the VraG T217I mutation (500× for BCFtools), and over 300× coverage was achieved within 10 min (Table S2).

**TABLE 3:** Mutation calls by BWA/BCFtools and Medaka for test strains EUH15 and 107^*a*^

Strain	BWA/BCFtools	Medaka
107 (VISA)	WalK A243T (26392)	WalK A243T (26392)
	GraR E15K (719060)	GraR E15K (719060)
	VraG T217I (718728)	VraS A314V (2026309)
	VraS A314V (2026309)	
EUH15 (VSSA)	None	None

a Mutations and reference coordinates are given relative to USA300 FPR3757.

**TABLE 4:** Minimum coverage required to call *walK*, *graR*, *vraG*, and *vraS* mutations with BWA/BCFtools and Medaka in VISA clinical strain or minimum coverage required to exclude mutations from wild-type samples for test strains EUH15 and 107^*a*^

Strain	Mutation	Coverage (×) for:	
		BWA/BCFtools	Medaka
107 (VISA)	WalK A243T (26392)	2	2
	GraR E15K (719060)	2	2
	VraG T217I (718728)	>500	
	VraS A314V (2026309)	2	5
EUH15 (VSSA)	WalK (26392)	20	0
	GraR (719060)	50	0
	VraG (718728)	0	0
	VraS (2026309)	80	0

a Mutation calls by BWA/BCFtools and Medaka given for test strains EUH15 and 107. Mutations and reference coordinates are given relative to USA300 FPR3757.

### Bioinformatic analysis: construction of a VISA-associated mutation database to enhance detection.

In order to broaden the scope of our method, we built a curated database of all VISA-associated mutations to make clinical VISA detection as sensitive and specific as possible. We noted the most mutations in Staphopia and synthetic mutation classes in the longest genes, such as 3,551-bp *rpoB* (10,653 synthetic mutations), 1,890-bp *vraG* (5,670 synthetic mutations), and 1,826-bp *walK* (5,478 synthetic mutations) (Fig. S2A). However, in the lab and clinical classes, *vraG* instead had the most mutations identified (229 lab and 217 clinical), followed by *graS* (112 and 108) and *vraF* (78 and 80). We also found no mutations in *sarA* called in either the lab or clinical data sets (Fig. S2A and B). We then annotated mutation calls with SnpEff to examine the distribution of mutation types (low, moderate, and high effect, corresponding to mainly synonymous, missense, and nonsense mutations, respectively) in each class. We found the most consistent mutation type distribution in the synthetic mutation class (20.50 ± 0.94% low, 73.43 ± 0.82% moderate, and 6.07 ± 0.72% high; mean and standard deviation across genes, respectively). Low-effect mutations were far more common in every other mutation class (47.76 ± 10.32% in the Staphopia class, 58.48 ± 27.24% in the lab class, and 70.86 ± 23.3% in the clinical class), by statistically significant margins in all cases (paired *t* tests; *P* = 1.103e-8 for Staphopia, 4.807e-5 for lab, and 2.81e-7 for clinical classes).

The critical control to evaluate our ability to discriminate VISA from VSSA based on this database was the overlap between these VISA database mutations and mutations called from parental N384 nanopore reads against the N384 reference genome. Such mutations would likely represent systematic sequencing errors that would be called false positives. To determine such overlap, we compared N384 and N384-3 sample mutation calls against the Staph_ASR reference (29) to each other and N384 or N384-3 unique calls to each of the four VISA mutation databases, reporting results in Table 5. We found that the number of shared mutations depends on the mutation detection method (BCFtools or Medaka). Neither detection method identified any mutations in the N384 parent that were also found in clinical or lab strains, but BCFtools did call 44 mutations shared with those found in Staphopia strains (9 low, 33 moderate, and 2 high). In N384-3, on the other hand, Medaka only detected the expected missense SNP in *walK* (646C>G, Q216E) and a conservative in-frame deletion in *vraR* (418T>TTTTTCATACGGTTACGCA, duplication of amino acids [aa] 134 to 139), both of which were also found in the lab-adapted strain mutation database. Meanwhile, BCFtools called over 100 mutations shared with clinical, lab, and Staphopia mutations. In each case, over 100 of these mutations were moderate effect (126 shared clinical, 131 shared lab, and 103 shared Staphopia mutations, respectively). This suggested that Medaka is more specific, while BCFtools is more sensitive in its mutation calling.

**TABLE 5:** Number of mutations called against Staph_ASR shared between N384 or N384-3 and VISA mutation databases (clinical, lab, and Staphopia) segregated by annotation type (low, moderate, or high effect) for each shared group^*a*^

Database	Predicted effect	N384		N384-3	
		BWA/BCFtools	Medaka	BWA/BCFtools	Medaka
Clinical	Low	0	0	7	0
	Medium	0	0	126	0
	High	0	0	6	0
Lab	Low	0	0	8	0
	Medium	0	0	131	2
	High	0	0	5	0
Staphopia	Low	9	0	41	0
	Medium	33	0	103	0
	High	2	0	1	0

a Mutations were annotated with SnpEff.

## DISCUSSION

Nanopore sequencing may revolutionize sequence-based antimicrobial resistance detection, as it allows very rapid data generation. Multiple studies have shown that nanopore sequencing can rapidly (15 min or less after sequencing begins) identify antibiotic resistance genes in plasmids (30) or antibiotic resistant strain lineages (21) and detect antibiotic resistance mutations in metagenomic sequencing from clinical samples (27). In this study, we explored PCR amplification of a set of genes known to be commonly associated with vancomycin-intermediate resistance. We took advantage of the long-read capabilities of this technology to reduce the number of amplicons to only 10 and showed that we could rapidly acquire 3,000× coverage from each. This approach offers the potential advantage of not needing to culture S. aureus from clinical samples, which could take 24 to 48 h. Indeed, the time lost to culturing has been suggested to be a cause of mortality itself, as mortality increases 10% with each hour in septic shock cases (31). Another advantage of the PCR approach is that the sequence data returned are only the portions of the pathogen genome known from prior studies to be associated with the phenotype, rather than metagenomic, or even whole-genome, data. Correspondingly, PCR provides higher coverage of these genes of interest than traditional whole-genome sequencing (WGS), making it possible to identify emerging resistant populations.

These results suggest that the assay has potential to identify VISA mutations in unknown clinical strains, thanks to extensive characterization of VISA in the clinic and in the lab and our curation of previously identified VISA mutations here. Our database also distinguishes known from suspected VISA because it also includes all possible nonsynonymous point mutations in VISA-associated genes, which could confer VISA. It is now possible to both potentially predict VISA and identify the mutational cause of the VISA phenotype in the same amount of time as culturing or less. We also found that it is possible to amplify commonly mutated genes conferring VISA with a small number of PCRs. Potentially, with high-quality host DNA depletion, we could amplify these genes without culturing. As in targeted nanopore sequencing assays in general, work still must be done to further improve (i) DNA extraction, (ii) PCR amplification, and (iii) basecalling.

In regard to the first area, we were unable to extract sufficiently high-quality DNA for downstream steps. Initial experiments yielded appropriate DNA template concentrations only for PCR from bacterial cultures themselves. We attempted spiking CD1 mouse blood with 1e-2 or 1e-4 CFU/mL N384 or N384-3, but in both cases, upon DNA extraction with the Qiagen DNeasy blood and tissue minikit, we only obtained nonspecific amplification with PCR for all amplicons, presumably from mouse DNA (data not shown). We also found that microwaving culture pellets resulted in DNA that was too fragmented to amplify our long regions of interest. This suggests that at least one culturing step (or host DNA depletion) may be necessary to isolate S. aureus for DNA extraction and PCR. We must reduce culturing time to as little as necessary to get isolated bacterial culture, followed by rapid (~1 h, including 30 min lysostaphin/lysozyme treatment) DNA extraction. The development of rapid approaches to extract bacterial DNA from clinical samples should be considered a critical research area. Recent advances have reduced human DNA 10-fold while maintaining bacterial DNA in a 7-h biopsy to pathogen identification protocol (32) and identified DNA extraction kits (Qiagen UCP pathogen minikit) that best depleted human DNA relative to Neisseria gonorrhoeae DNA in clinical urine samples (33).

The PCR primers amplified all 10 regions in simplex reactions that took about 2 h, but further development would be necessary for optimization. Ideally, these reactions should be multiplexed in as few tubes as possible. The solution may be to explore using a higher number of shorter amplicons. This scheme may also be more robust to lower S. aureus DNA template yield and quality. The limits of detection of the PCR system need to be thoroughly explored on spiked and real clinical samples. At the very least, PCR and sequencing should be able to verify the presence of S. aureus in the clinical sample to a certain CFU/mL titer. Though we acknowledge that S. aureus DNA could persist in patient samples longer than viable S. aureus, together with clinical signs and symptoms, nanopore sequencing evidence for VISA could aid clinical decision making.

Regarding sequencing time, we need to determine the minimum number of reads necessary to call a mutation in our mutant/parent mixtures by randomly subsampling various numbers of reads and repeating our variant-calling pipelines. We also need to see whether fast basecalling is sufficiently accurate to call VISA mutations, as we used the high-accuracy, neural network-dependent Bonito basecaller (34) with external graphics processing unit (GPUs) after sequencing. We attempted to speed up alignment with Medaka, but our standard protocol (BWA/BCFtools) sensitively and completely called known VISA mutations. Nanopore technology currently has a higher sequencing error rate (~3 to 5% average per read per base for the newest versions) than other methods. In addition, these sequencing errors are often systematic, focused in loci such as homopolymer regions. The critical problem to overcome for these nanopore applications is distinguishing sequencing errors from causative resistance mutations when analyzing sequence traces. Medaka, a rapid neural network-based alternative to standard alignment and variant calling (35), proved ineffective both in sensitively detecting mutations in the mutant/parent mixtures and in comprehensive detection of the strain 107 VISA mutations. Thus, our method will be refined further to amplify all regions together through a multiplex PCR and detect VISA mutations in the shortest possible time through machine learning methods.

Future work will involve validating this assay further in more genetically diverse VISA and VSSA strains and further correcting for nanopore sequencing errors. Full validation of the method would require a thorough comparison with real clinical phenotypes, which is not practical given the rarity of VISAs. The construction of a VISA mutation database for comprehensive mutation detection in unknown samples, including lab-evolved, clinical, and predicted VISA mutations in one database, has already provided insights into the mutation call sensitivity and specificity of our assay. We anticipate this database should make detection possible across diverse strains and serve as a resource for VISA resistance characterization and population genetics more generally. The critical control we must resolve is sequencing errors that overlap with mutations in this database. We have assessed this by comparing N384 parental mutations with those in our database. We expect that further future improvements in nanopore basecalling and mutation calling (with Medaka) will translate into reductions in false-positive VISA calls in our assay.

## MATERIALS AND METHODS

### Overview of the nanopore sequencing VISA detection pipeline.

**FIG 3:**
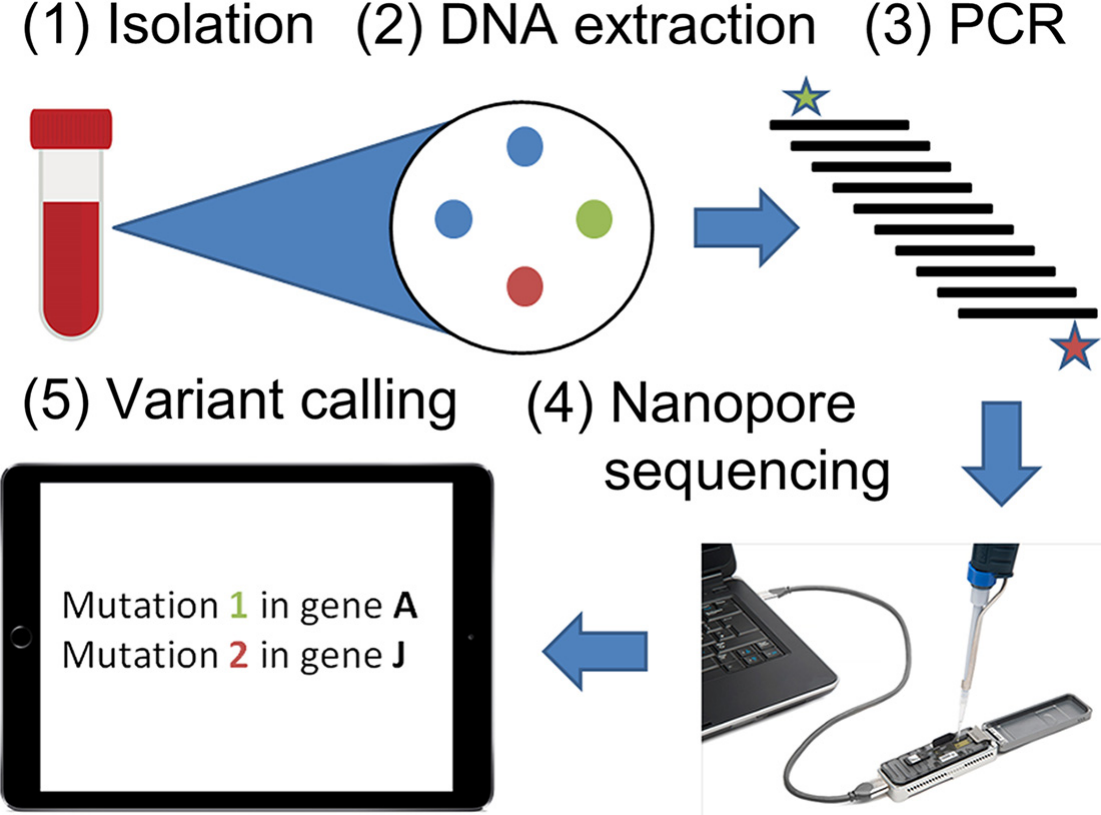
Overview of the VISA amplicon sequencing process, from sample isolation to SNP calling and comparison to known VISA mutations. Steps 1 to 5 represent sample isolation, DNA extraction, PCR, nanopore sequencing, and variant calling, respectively. In step 1 bacteria are grown in a blood bottle (left), from which multiple colonies are cultured (right), including both the two individual VISA mutants (red and green) and the VSSA parental strain (blue). Step 2 represents DNA extraction from the pooled colonies cultured at the end of step 1, while step 3 represents PCR amplification of VISA amplicons from the extracted bacterial DNA. Step 4 shows the loading of an amplicon nanopore library on an Oxford Nanopore MinION instrument. Finally, step 5 shows the calling of the two VISA mutations in the sequenced nanopore library against reference gene sequences. Two VISA mutations are illustrated in red and green (steps 2, 3, and 5), while their parental strain is in blue.

We developed a strategy combining DNA extraction from an S. aureus isolate, PCR amplification of S. aureus genes often implicated in VISA, and nanopore sequencing of these amplicons to identify mutations. Our proposed pipeline is outlined in Fig. 3. S. aureus may be isolated either directly (isolation from culturing) or indirectly (metagenomic DNA extraction) from a clinical sample such as a blood bottle. S. aureus populations cannot be assumed to be clonal; instead, they may include a mixture of the VSSA parental strain and at least one VISA mutant, if not more. Challenges must be addressed in five general areas: (i) sample isolation, (ii) PCR of multiple unlinked genomic regions, (iii) sequencing, (iv) mutation calling and calling of VISA/VSSA based on mutation patterns, and (v) bioinformatic determination of whether potential VISA mutations had been previously characterized. Sample isolation must both produce enough DNA for PCR and remove any contaminating nonbacterial DNA that would lead to subsequent nonspecific PCR. The PCR method must amplify each fragment to a similar relative abundance even from the lowest DNA input. Mutation calling must distinguish sequencing errors from VISA-causing mutations and allow for detection of subpopulations of unfixed VISA mutations. False-positive errors would suggest spurious emergent resistance in clinical samples; false-negative errors would miss a true resistant subpopulation. We thus compared multiple mutation calling approaches to reduce false positives and negatives as well as increase detection sensitivity.

### Strain selection and media.

Our strain panel consisted of MRSA parent strain (N384), its lab-selected VISA mutant (N384-3) (36), clinical VSSA (EUH15), and clinical VISA (107) strains. N384-3 was selected from N384 through stepwise evolution in vancomycin up to 8 μg/mL. Strains and associated metadata (vancomycin MICs) are listed in Table 6. All strains were grown on tryptic soy agar (TSA) or in tryptic soy broth (TSB) at 37°C with 225 rpm agitation for liquid culture.

**TABLE 6:** Strains used in this study

Strain	Description	Reference
N384 (NRS384)	Community-associated USA300 MRSA strain (CC8)	36
N384-3	Laboratory selected VISA mutant in N384 background; mutation *walK* 646C>G Q216E	36
EUH15	VSSA clinical isolate (CC5); vancomycin MIC is 1 μg/mL	6
107	VISA clinical isolate (CC5); vancomycin MIC is 4 μg/mL	6

### Preparation of mutant/parent mixtures (cell and DNA) for evaluating limit of detection.

To evaluate the limit of mutation detection, mutant and parent sequences were mixed together in five ratios from 100% N384-3 mutant to 100% N384 parent (100% N384-3, 90% N384-3; 10% N384, 50% N384-3; 50% N384, 10% N384-3; 90% N384, 100% N384). Either turbid bacterial cultures or amplicon DNA volume were mixed in these ratios (total amplicon volume of 100 μL; 1 mL culture volume; 3e-9 CFU/mL). DNA (including amplicon) concentrations were measured with a Qubit double-stranded DNA (dsDNA) broad range (BR) assay kit (Invitrogen). DNA was extracted from cell mixtures with a modified Qiagen DNeasy blood and tissue minikit. Cultures were pretreated for 30 min with 0.2 mg/mL lysostaphin and 1 mg/mL lysozyme at 37°C to cleave S. aureus cell walls, and then DNA was extracted following the manufacturer’s directions.

### Amplifying VISA-associated loci.

VISA amplicons represented regions from the USA300 FPR3757 genome containing VISA-associated genes or clusters of such genes (e.g., the *walRK* two-component system) and 1,000 bp adjacent sequence on either side (Table S1). These primers were confirmed to be robust by *in silico* alignment against the Staphylococcus aureus Artificial Sequence Reference from Staphopia (29) and testing for specificity against S. aureus and S. epidermidis with Primer-BLAST (37) (no hits were found for S. epidermidis). These regions were amplified through PCR from N384 parent/mutant DNA mixture templates (0.2 ng/μL). All regions were amplified using NEB Q5 high-fidelity 2× master mix and the following PCR program: 98°C for 30 s (initial denaturation); 98°C for 10 s, 65°C for 30 s (annealing), and 72°C for 3 min 30 s (35 cycles; extension for up to 6.5 kbp sequence); 72°C for 2 min, final extension; then hold at 4°C.

### Amplicon nanopore sequencing.

VISA amplicons were pooled to a total of 1 μg for sequencing (equal quantities of each amplicon). VISA amplicon libraries were generated using the 1D ligation sequencing kit (SQK-LSK109; Oxford Nanopore) modified for amplicon sequencing. Amplicon DNA (0.2 pmol) was ligated to adapters to bias ligation reactions toward individual amplicons. Libraries were sequenced on Flongle FLO-FLG001 flow cells (Oxford Nanopore) on a MinION instrument. Read data (event-level, FAST5 format) were collected on a PC using MinKNOW software (Oxford Nanopore).

### Basecalling, alignment, and variant calling.

For *de novo* basecalling, Bonito (version 0.3.6) (34) was used with nucleotide output stored as a FASTA file. For detecting nucleotide changes compared to a reference (mapping), all basecalled reads called by the bonito software (including EUH15 and 107 reads) were aligned against the USA300 FPR3757 reference using BWA (version 0.7.17; parameter -x ont2d) (38). Variants were called with BCFtools (version 1.11; mpileup/call on the resulting read alignment with consensus and multiallelic variant calling) and Medaka (version 1.6.0; default parameters, reads against USA300 FPR3757 reference), with results saved in a VCF file. Variants were further filtered down to biallelic sites with BCFtools view. The consensus sequence was determined using SAMtools mpileup (-B option selected to cancel base alignment quality assessment) and BCFtools (-c option for consensus determination). Variants were compared to the known N384-3 VISA mutations (*walK* 646C>G) in the case of N384 parent/mutant mixtures and known, previously identified VISA-associated mutations in the case of EUH15 and 107 test strains.

### *In silico* read simulation and mutation frequency analysis.

*In silico* amplicon sequence mixtures (300,000 reads in each case; 100% N384-3, through 100% N384 in 10% increments of each) were simulated using NanoSim-H (version 1.1.0.4) (39, 40) using default parameters from the wild-type (N384) and VISA mutant (N384-3) amplicon sequences concatenated into a single FASTA sequence (predicted amplicons from primers in Table S1). Amplicon reads were simulated with an error profile trained from Bonito-basecalled N384 parent amplicons (sequenced from amplicon DNA rather than cell mixture series). A total of 300,000 reads were simulated in each case. These read mixtures were then used together with cell and DNA mixture reads to determine how VISA mutation frequency correlated between variant calling (observed mutation frequency) and VISA proportion (predicted VISA mutation frequency) and to determine the detection threshold without the use of Medaka. In order to address these questions, simulated and real (cell or DNA) mixtures were aligned against the USA300 FPR3757 (N384) reference using BWA (version 0.7.17; parameter -x ont2d) (38) and analyzed for variant counts with SAMtools (41) mpileup (-B option selected to cancel base alignment quality assessment). Mpileup output was further processed into a table with counts of every base, positive strand matches (.), negative strand matches (,), and overall mapped read coverage at each amplicon site. Overall nonreference nucleotide frequency was calculated for every mapped amplicon nucleotide to generate an overall distribution with a mean and standard deviation. For each proportion of mutant, the detected mutation (*walK* 646C>G at USA300 FPR3757 position 26311) is proportional to the mapped coverage and the Z-score. The detection limit was calculated as the extrapolated observed mutation proportion for which the Z-score exceeded 2 (2 standard deviations above the mean to give two-tailed 95% confidence).

### Evaluating minimum coverage necessary for VISA mutation detection.

We randomly subsampled three types of nanopore amplicon data (*in silico* simulated, cellular, and DNA parent/mutant mixtures) plus the additional test VSSA (EUH15) and VISA (107) strains to sequence coverages ranging from 2 to 500× in order to detect the minimum sequence coverage necessary for correctly detecting VISA mutations. We subsampled reads with seqtk (version 1.3-r106). Subsampled reads were aligned against the USA300 FPR3757 reference with BWA, and mutations were called with BCFtools and Medaka as previously described. Subsampled read mutation calls were then compared to the expected mutation (*walK* 646C>G at USA300 FPR3757 position 26311). The minimum coverage necessary to call this mutation was estimated as the minimum coverage for which the expected mutation was detected in the mutation calls. Coverage achieved at time points throughout each sequencing run (at 10-min intervals) was evaluated with NanoPlot (version 1.30.1) (42).

### Constructing a VISA mutation database.

A database of VISA mutations was constructed from four levels of information: (i) lab-evolved VISA mutations identified through successive selection of mutants in increasing vancomycin concentrations, (ii) VISA-associated mutations in clinical strains, (iii) predicted VISA-causing mutations detected in our database of 40,000+ S. aureus genomes, and (iv) simulated mutations in VISA-associated genes expected to cause VISA. This database was constructed by (i) collecting mutations identified in our laboratory VISA evolution studies (36), (iii) collecting those previously identified in clinical VISA strains in our lab (6), (iii) finding nonsense or missense mutations relative to the N315 reference in our Staphopia database (29), and (iv) performing a saturating mutagenesis of the genes in our amplicons and identifying all nonsense or missense mutations predicted to confer VISA. Previously screened clinical strains were selected as VISA if they showed an MIC of over 4 μg/mL with any MIC determination method used (broth microdilution [BMD], Etest, or population analysis profile-area under the curve [PAP-AUC]). Short reads for lab-adapted VISA strains, clinical VISA strains selected based on the stated MIC criteria, or all strains in the Staphopia database were aligned against the Staph_ASR reference with BWA. Mutations were called against the Staph_ASR reference (classes 1 and 2, described previously) with Snippy (version 4.6.0) (43), selected from the Staphopia database (29), or simulated in the Staph_ASR reference (class 4) with the syntheticVCF tool compiled in C (44). Mutations called per strain in classes 1 to 3 were then merged into a nonredundant set per class with BCFtools (version 1.11) merge. Variants were filtered for VISA gene coordinates with VCFtools (version 0.1.16), and variant effects were annotated with SnpEff (version 5.0e). The Staph_ASR genome was annotated with Bakta (version 1.4.0) (45) for use in SnpEff.

### Comparison of nanopore sample mutation calls against VISA mutation database.

To evaluate sensitivity and specificity of mutation calling from our nanopore samples (N384 and N384-3), we aligned N384 and N384-3 (100% of each in the amplicon DNA series) nanopore reads against the Staph_ASR genome with BWA and called variants with BCFtools call or Medaka. We then identified Medaka- or BCFtools-called mutations specific to N384 or N384-3 by comparing N384 and N384-3 mutation calls with BCFtools isec. N384- or N384-3-specific mutations were then compared to lab, clinical, and Staphopia mutation databases also with BCFtools isec. Mutations found to be shared between N384- or N384-3-specific mutations and each database were then annotated with SnpEff and classified by mutation type (low, moderate, or high effect).

### Data availability.

Raw sequencing data generated from the MinION instrument were deposited in the NCBI Sequence Read Archive (SRA) under BioProject accession PRJNA863907.
