# Supplementary material for: Development of an Amplicon Nanopore Sequencing Strategy for Detection of Mutations Conferring Intermediate Resistance to Vancomycin in Staphylococcus aureus Strains
Source: Microbiol Spectr. 2023 Jan 23;11(1):e02728-22. doi: 10.1128/spectrum.02728-22 (PMC9927139; doi:10.1128/spectrum.02728-22)
Supplement: Supplemental file 1 — Supplemental material. Download spectrum.02728-22-s0001.pdf, PDF file, 0.2 MB [file spectrum.02728-22-s0001.pdf]

Supplementary Information for Development of an amplicon nanopore sequencing strategy for detection of mutations conferring intermediate resistance to vancomycin in *Staphylococcus aureus* strains

Abraham G. Moller[1,2], Robert A. Petit III[1,3], Michelle H. Davis[1], and Timothy D. Read[1]

[1]Division of Infectious Diseases, Department of Medicine, Emory University, Atlanta, GA, 30322
[2]Microbiology and Molecular Genetics (MMG), Graduate Division of Biological and Biomedical Sciences (GDBBS), Emory University, Atlanta, GA, 30322
[3]Theiagen Genomics, Highlands Ranch, CO, 80129

Supplementary Figure S1: Amplification of 10 VISA regions from N384 mutant template DNA extracted from pure culture. From left to right, the amplicons are *walRK*, *rpoB*, *graRS*, *vraFG*, *stpI*, *vraRS*, *agrA*, *sarA*, *clpP*, *ccpA*, *prsA*, and *yvqF*. The NEB 1 kb Plus ladder is shown for reference at left. The largest marker on the ladder is 10 kb and the smallest is 0.1 kb. The ladder and all amplicons were run at 110V for 30 minutes.

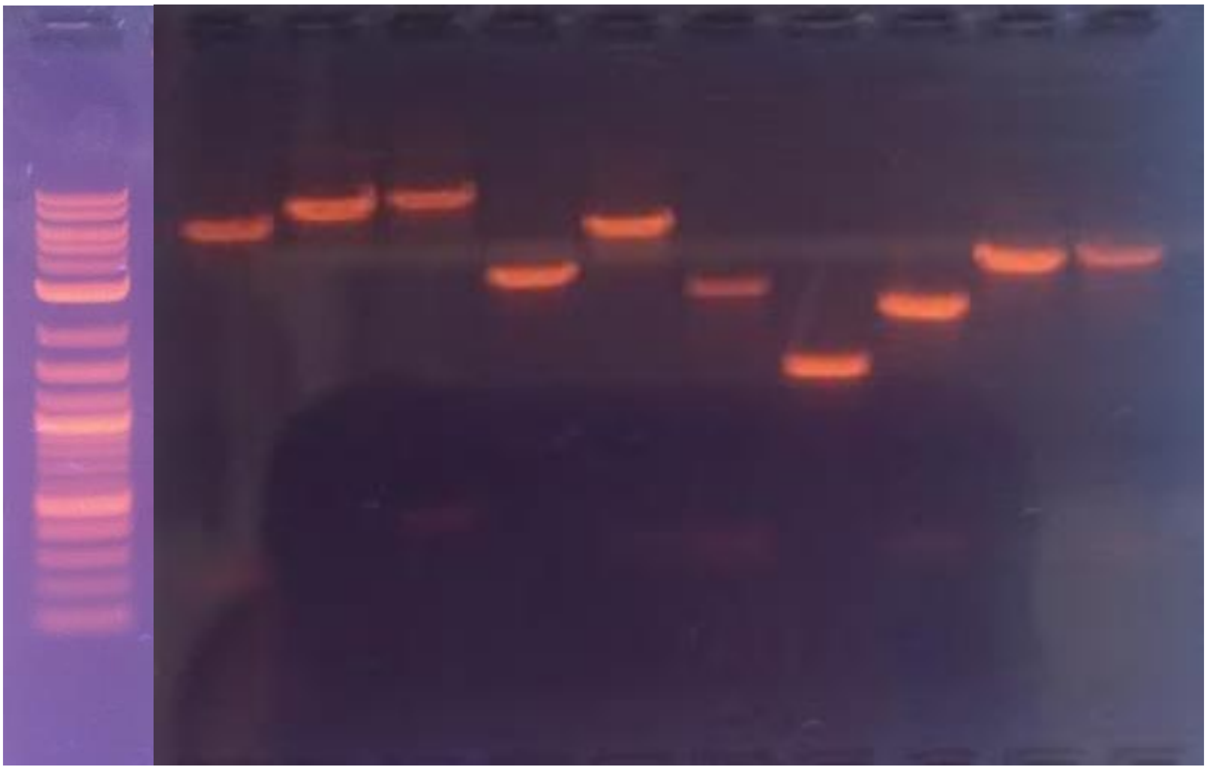

Supplementary Figure S2: Distributions of annotated variant types in VISA mutation database. Variants in 16 known VISA-associated genes were called in clinical VISA and lab-adapted VISA short read data against the Staph_ASR reference with snippy and annotated with SnpEff. Variants in our curated database of 40,000+ *S. aureus* genomes and all possible simulated mutations in the VISA-associated genes were also annotated with SnpEff. Proportions of high, moderate, and low effect mutations are marked in red, blue and green, respectively. Either raw mutation counts (A) or proportions (B) are shown for each gene and mutation effect class, respectively.

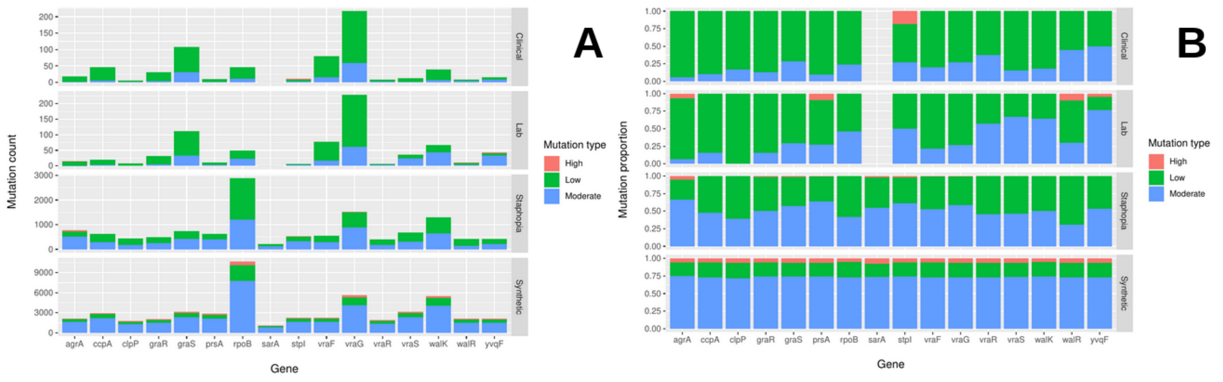

Supplementary Table S1: Primers used for amplifying each of 10 regions containing genes likely to contain VISA-conferring mutations, melting temperatures calculated for each primer with the NEB Tm calculator, and amplified region sizes.

ID 1	Primer 1 sequence	Tm 1	ID 2	Primer 2 sequence	Tm 2	Amplicon size
walRK F	GATTCCCGTCGAGACCGTAC	67	walRK R	CGCTTCATCTTCGGACAGGT	67	4546
rpoB F	GCGAATTGCCGATGTTGGTT	67	rpoB R	TGCAACGAATTGACCTGGGT	67	5551
graRS-vraFG F	GCTTTGAAGTTGACTGCCGG	67	graRS-vraFG R	AGCAGCACGATCCAGATTGA	66	6493
stpI F	AAGCTTACACGCCGCAAAAG	67	stpI R	CTGATGATGAGCAGGCCCAT	67	2743
vraRS-yvqF F	CATATGGCAGTATCGCGGGT	67	vraRS-yvqF R	ATGGGCTTTTGAAACGAGCG	66	4363
agrA F	GAAGATGACATGCCTGGCCT	68	agrA R	TTGATACAACTGGGGCAGGG	67	2414
sarA F	GCGGTGGCAATTCGTTCATT	67	sarA R	TCGGGCAAATGTATCGAGCA	67	2373
clpP F	CGACATTGCGGGATTCTCTG	66	clpP R	TTGTCATCGGTCGTTTCGGT	67	2597
ccpA F	CTCAACCTGGTCGAGCAAGT	67	ccpA R	TTGCACTTAGTGATGCGGGT	67	2944
prsA F	ATCCCCACTTTCGCGTTTCA	67	prsA R	GCACCTTTATCACCGGCAGA	68	2960

Supplementary Table S2: Coverage at earliest time point (10 minutes) as determined from NanoPlot sequence yield over time analysis.

Sample/strain	Type	Time (min)	Yield (Gb)	Genome size (kb)	Coverage
EUH15	Culture	10	0.0174	36.984	469.826
107	Culture	10	0.0176	36.984	474.744
N384 100	Culture	10	0.0194	36.984	523.889
N384 90	Culture	10	0.0134	36.984	363.160
N384 50	Culture	10	0.0164	36.984	444.760
N384 10	Culture	10	0.0141	36.984	379.901
N384 0	Culture	10	0.0215	36.984	580.936
N384 100	DNA	10	0.0133	36.984	359.094
N384 90	DNA	10	0.0116	36.984	314.859
N384 50	DNA	10	0.0191	36.984	516.244
N384 10	DNA	10	0.0166	36.984	448.888
N384 0	DNA	10	0.0112	36.984	301.671
